# Piston-Based Material Extrusion of Ti-6Al-4V Feedstock for Complementary Use in Metal Injection Molding

**DOI:** 10.3390/ma15010351

**Published:** 2022-01-04

**Authors:** Lennart Waalkes, Jan Längerich, Philipp Imgrund, Claus Emmelmann

**Affiliations:** 1 Fraunhofer Research Institution for Additive Manufacturing Technologies IAPT, Am Schleusengraben 14, 21029 Hamburg, Germany; jan.laengerich@iapt.fraunhofer.de (J.L.); philipp.imgrund@iapt.fraunhofer.de (P.I.); 2 Institute of Laser and System Technologies iLAS, Hamburg University of Technology TUHH, Denickestr. 17, 21073 Hamburg, Germany; c.emmelmann@tuhh.de

**Keywords:** additive manufacturing, material extrusion, Ti-6Al-4V, feedstock, metal injection molding, green parts

## Abstract

Piston-based material extrusion enables cost savings for metal injection molding users when it is utilized as a complementary shaping process for green parts in small batch sizes. This, however, requires the use of series feedstock and the production of sufficiently dense green parts in order to ensure metal injection molding-like material properties. In this paper, a methodological approach is presented to identify material-specific process parameters for an industrially used Ti-6Al-4V metal injection molding feedstock based on the extrusion force. It was found that for an optimum extrusion temperature of 95 °C and printing speed of 8 mm/s an extrusion force of 1300 N ensures high-density green parts without under-extrusion. The resulting sintered part properties exhibit values comparable to metal injection molding in terms of part density (max. 99.1%) and tensile properties (max. yield strength: 933 MPa, max. ultimate tensile strength: 1000 MPa, max. elongation at break: 18.5%) depending on the selected build orientation. Thus, a complementary use could be demonstrated in principle for the Ti-6Al-4V feedstock.

## 1. Introduction

Metal injection molding (MIM) is a production technology that is primarily suitable for high production volumes since molds are required that only amortize with increasing quantities [1,2]. During injection molding, a feedstock is injected into the mold to form a so-called green part, in which a metal powder (solids loading between 50 and 67 vol% [3]) is bound within a polymer matrix [4]. After molding the green part, the polymer components are successively removed in a debinding step, followed by a final heat treatment to sinter the remaining metal powder into a nearly full dense metal part [1]. The additive manufacturing (AM) of green parts can thus lead to time and cost savings in metal injection molding when it comes to functional prototypes, custom-made or complex parts with hollow structures since no molds are required [5]. For this field of application, piston-based material extrusion (PEX) was introduced as a new complementary AM process for MIM users by combining the main advantages of the polymer-based AM processes fused filament fabrication (FFF) and fused granular fabrication (FGF) [6].

Due to its ease of operation and low machine costs, FFF is already well studied for printing green parts from powder-binder formulations similar to MIM feedstock [7,8,9,10,11,12]. However, typical MIM feedstock formulations must be adapted to filament requirements such as a sufficient flexibility for spooling by adding, for instance, elastomers [13] or amorphous polyolefins [14]. To keep changes to debinding and sintering as low as possible, the use of highly filled filaments is thus not preferable for the intended complementary green part production [6]. Screw-based extrusion, on the other hand, is suitable for this purpose, as it allows conventional MIM feedstock to be processed [5,15,16,17,18]. Yet, machine costs are typically about ten times higher than FFF printers, since print heads are equipped with complex and expensive screw geometries [19,20].

To overcome the main disadvantages of FFF and FGF regarding the additive manufacturing of green parts, PEX enables the processing of MIM feedstock at machine costs typical for FFF printers. This opens up application areas especially in the medical, aerospace, and consumer sector for small batch sizes (<100 p.a.) (cf. [21]). In the medical and aerospace sector, for example, the rapid availability of functional prototypes can significantly reduce the MIM product development process. Furthermore, consumer goods such as high-end bicycle parts or mountaineering equipment can be produced on customer request. Such low volumes are not economical due to the mold production in MIM, which enables new business areas for MIM users towards low volumes through the complementary use of PEX. To ensure low costs per part, the extrusion process for PEX takes place—analogous to low-cost FFF printers—with the aid of a stepper motor-driven gear drive. This allows the use of low-cost FFF product architectures in terms of software and hardware. In addition, pistons are easier to clean than complex screw geometries, which allows quick material changes [22].

However, to enable complementary use in MIM, green parts printed with PEX must have comparable properties to injection-molded parts. Therefore, under-extrusion must be avoided. Generally, under-extrusion is accompanied by distinct rhomboid voids between adjacent extrusion paths [23], which reduces the density of the green part [24]. These defects are then transferred to the sintered part, resulting in low-density values and poor mechanical properties. In PEX, the state of compaction has a significant influence on the extrusion process—under-extrusion in particular. If the feedstock is not sufficiently compacted before the print job starts, the molten material will be compacted during the printing process. The volume flow extruded through the nozzle is consequently lower than required, resulting in under-extrusion and low-density green parts. Therefore, to enable a complementary use of PEX, measurement of the state of compaction is needed to avoid under-extrusion.

In this work, the force acting against the piston rod during extrusion is introduced as a parameter for quantifying the compaction state of the molten feedstock inside the cylinder. For this purpose, an existing PEX system is equipped with strain gauges and calibrated on the basis of a theoretical model of piston extrusion. With the help of this in-process measured value, a methodical approach is presented which allows material-specific process parameters to be derived for a granular Ti-6Al-4V feedstock that is used in industrial MIM process chains. The aim of these material-specific process parameters is to produce dense green parts without rhomboid voids in order to achieve comparable material properties in the sintered state. First, mass flows are determined experimentally for a defined time, temperature, and force interval. These allow to calculate force- and temperature-specific printing speeds at which dense green parts can be expected for PEX. Validation of the calculated printing speeds is performed by printing tests, in which the green parts are evaluated with respect to their density by means of fracture surface analysis. Based on this, an extrusion force is identified for an optimum printing speed and extrusion temperature for the Ti-6Al-4V feedstock used. Test specimens are then printed using the process parameters found and debound and sintered in the same way as injection molded parts. The resulting sintered parts are finally analyzed in terms of part density and tensile properties and compared to corresponding MIM values.

## 2. Materials and Methods

### 2.1. MIM Feedstock

**Figure 1:**
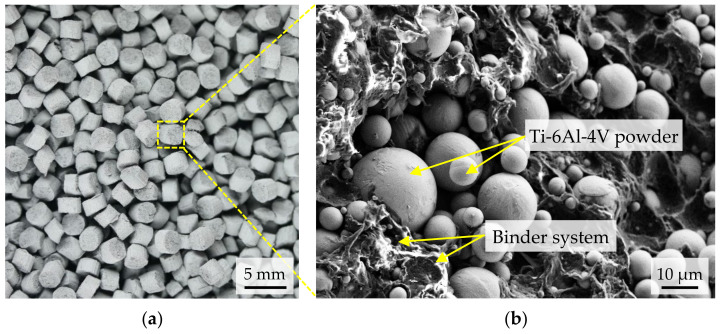
(**a**) Macro shot of granular Ti-6Al-4V feedstock; (**b**) SEM image of feedstock ingredients.

With regard to a complementary use in MIM, the processing of unmodified MIM feedstock is necessary in order to avoid adjustments during debinding and sintering (see Section 2.3). For this reason, a commercially available Ti-6Al-4V MIM feedstock was used for this work [24]. The feedstock is uniformly granular (s. Figure 1a), containing 66 vol.% spherical Ti-6Al-4V powder with a particle size distribution of D_90_ = 19 μm. The Ti-6Al-4V powder is embedded in a proprietary binder system with paraffin wax as the main polymer component, as can be seen in the SEM image (Leo, Gemini 1530) in Figure 1b. The calculated density of the multi-component feedstock system is 3.23 g/cm^3^. In order to remove the main polymer component, a solvent-based debinding process is carried out first. The residual polymer components are then thermally removed and the remaining Ti-6Al-4V powder is sintered to a nearly dense metal part. The theoretical density of the used powder is 4.43 g/cm^3^ [25].

### 2.2. Piston-Based Material Extrusion

**Figure 2:**
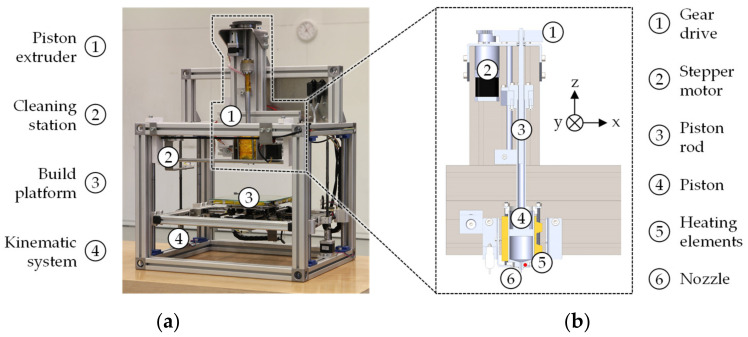
(**a**) Image of the PEX system used; (**b**) cross-section of the piston extruder as CAD design.

In order to process MIM feedstock with cost-efficient hardware and software from FFF printers, the PEX (also known as piston-based feedstock fabrication, PFF) system in Figure 2a is controlled via steps per millimeter according to an extrusion model [6]. To ensure a precise control per step, the piston is driven by a stepper motor (NEMA 17), which is reduced (130:1) by two gears, as can be seen in the CAD design in Figure 2b. The granular MIM feedstock is filled between the piston and the nozzle (capacity: 105.3 cm^3^) and completely melted by the heating elements. Subsequently, the molten material is compacted and extruded through the nozzle by a downward movement of the piston at a defined speed. The extruded feedstock is deposited on a print platform according to the cross-section of the part to be generated. A kinematic system moves the print platform in x-, y-, and z-direction so that acceleration of the extruder’s high mass is avoided. After depositing a layer, the print platform is lowered by one-layer height and new layers are deposited until the green part is completely built up.

#### 2.2.1. Extrusion Model

**Figure 3:**
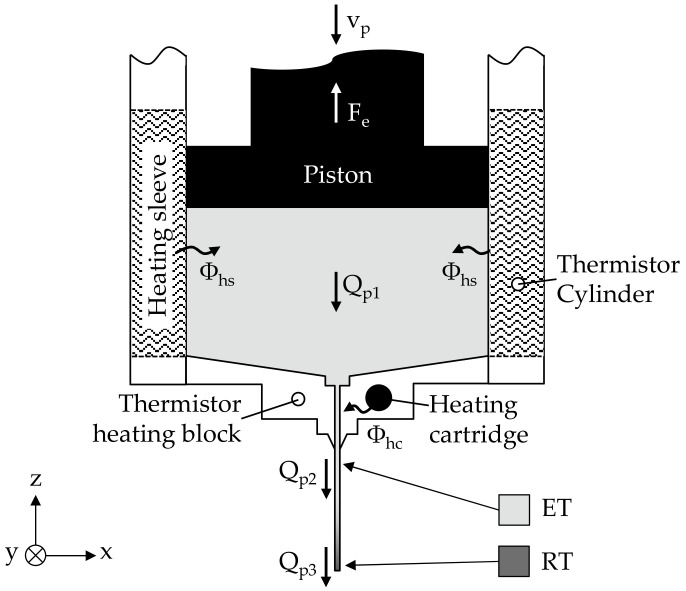
Extrusion model for piston-based material extrusion.

During piston extrusion, the granular MIM feedstock between the piston and the nozzle is heated to an extrusion temperature (T) and melted with the aid of the heat flow of two heating sleeves (Φ_hs_) and a heating cartridge (Φ_hc_); both heat flows being controlled via thermistors. By moving the piston downwards and thus pushing the molten feedstock out of the nozzle, an extrusion force acting on the piston rod can be measured, as shown in Figure 3. This extrusion force correlates with the printing speed and extrusion temperature set in the slicing software (Slic3r, version 1.3.0).

For a step-controlled extrusion process analogous to FFF, the number of steps for one millimeter of piston feed is defined in the firmware (Marlin, version 1.1.9.1). Based on this, the slicing software calculates the required volume flow (Q_s_) at room temperature (RT) according to Equation (1):

Q_s_ = v∙A (1)

v: Printing speed [mm/s]
A: Approximated cross-sectional area of extrusion path [mm^2^]

In contrast to FFF—and assuming mass conservation—in PEX the input volume flow Q_p1_ is equal to Q_p2_ exiting through the nozzle since both are displaced at extrusion temperature (ET). The essential prerequisite for satisfying Equation (2) is an extrusion force that ensures appropriate compaction of the molten feedstock for a defined speed and temperature. To control the piston extrusion process with standard FFF slicing software, Q_p1_ and Q_s_ are equated by definition as described in Equation (4) using Equation (3). This is based on the analogy that for both PEX and FFF the molten material is extruded by a piston since the solid filament above the melting zone also acts as a piston that forces the already molten material through the nozzle.

Q_p1_ = Q_p2_ = q_m_/ρ_ET_ (2)

Q_p1_ = v_p_∙A_p_ (3)

Q_p1_ = Q_s_ → v_p_ = v∙A/A_p_ (4)

Q_p1_ = q_m_/ρ_ET_ > q_m_/ρ_RT_ = Q_p3_ (5)

q_m_: Mass flow [g/s]
ρ: Feedstock density at RT and ET [g/mm^3^]
v_p_: Piston speed [mm/s]
A_p_: Cross-sectional area of piston [mm^2^]

Yet, as can be derived from Equation (5), this analogy has the consequence that the cooled output volume flow Q_p3_ is slightly lower than Q_p1_—and Q_s_ by definition—due to the temperature-related density difference. During cooling from Q_p2_ to Q_p3_, the specific volume decreases, and the specific density increases [26]. Nevertheless, for the majority of MIM feedstock systems, due to their low-melting waxes as binder components [4], it can be assumed that this difference has no significant influence on the green part density. This is because the melt zone in FFF printers is significantly smaller than PEX so that more energy must be applied to convert the material into a sufficiently low-viscosity state. Thus, smaller differences between RT and ET can be expected for PEX accompanied by a smaller decrease in specific volume during cooling. The green part to be printed is additionally heated above room temperature by a heated print bed. Consequently, no under-extrusion in the form of rhomboid voids between adjacent extrusion paths is to be expected, since small temperature-related differences in the output volume flow are compensated by shrinkage. A prerequisite for this, however, is sufficient compaction by a viscosity-related extrusion force as a function of printing speed and extrusion temperature.

#### 2.2.2. Extrusion Force

**Figure 4:**
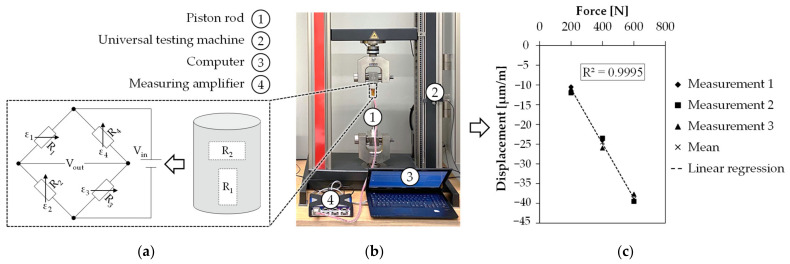
(**a**) Wheatstone full bridge circuit with four active strain gauges; (**b**) experimental setup for calibration; (**c**) results of calibration.

To measure the extrusion force F_e_, the force acting on the piston rod during the extrusion of volume flow Q_p1_ was measured. For this, strain gauges (Kyowa Electronic Instruments, KFG-5-120-C1-16L1M2R) were attached to the piston rod in a full-bridge circuit. The full-bridge circuit consists of two pairs of strain gauges, each with two measuring strips, which were mounted opposite each other on the piston rod. The measuring strips themselves are offset orthogonally to each other, as can be seen in Figure 4a. Such a setup is particularly suitable for measuring a compression load, whereby temperature influences are compensated for as far as possible [27].

To calibrate the selected strain gauge arrangement, the force-induced displacement was determined experimentally with the setup shown in Figure 4b. For this, the piston rod was clamped in a universal testing machine (ZwickRoell, Z010) and connected to a computer via a measuring amplifier (HBM, QuantumX MX840B). During calibration, the piston rod was subjected to a defined force (200 N, 400 N, 600 N) in compression, with three measured values being recorded per force. As can be seen in Figure 4c, the measurement results indicate a strong linear relationship with a coefficient of determination of R^2^ > 99%. Based on the measured linearity, the strain gauges were calibrated to convert the displacement measured during extrusion into the extrusion force F_e_. For plotting F_e_, the proprietary software Catman Easy from HBM was used. Before plotting, the piston was placed in the upper part of the cylinder and heated together with the feedstock to extrusion temperature for at least 30 min. Care was taken to ensure that no external forces acted on the piston. The measured force was then zeroed and the piston is moved down to compact the feedstock initially with a temperature- and printing speed-specific extrusion force, followed by starting the print job.

### 2.3. Debinding and Sintering

**Figure 5:**
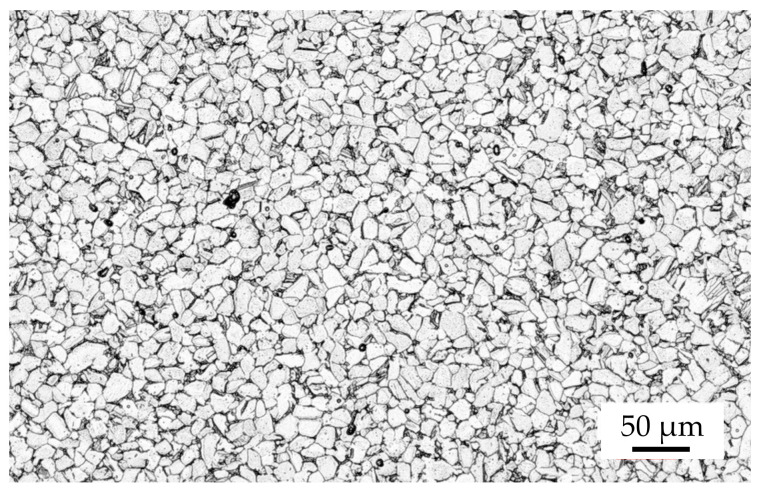
Microstructure of used Ti-6Al-4V feedstock as-sintered.

The typical chemical composition of sintered titanium parts produced from the used feedstock can be found in Table 1. In general, titanium is characterized by a high affinity to interstitial elements such as oxygen or carbon. Oxygen in particular is taken up preferentially and has the greatest influence on the mechanical properties [28]. Therefore, attention must be paid to oxygen uptake during debinding and sintering. Both are performed externally at Element22 GmbH, so not all process parameters are known. However, in the sense of complementary use, the additively manufactured green parts were debound and sintered with the same process parameters as injection molded green parts. In order to first remove the paraffin wax from the green parts, a solvent debinding step was carried out in hexane at 40 °C for 18 h. The final sintering process was conducted in high vacuum for less than 3.5 h below the beta transus temperature (<1100 °C) [29]. Typically, Ti-6-Al-4V is sintered above the beta transus temperature at about 1300 °C in the single-beta phase region [28]. The significantly lower sintering temperature promotes a fine-grained microstructure, as can be seen in Figure 5 as an example. This is made possible by the combination of fine powder (maximum particle size < 25 µm) and sintering in the alpha-beta phase region [21].

### 2.4. Methodology

**Figure 6:**
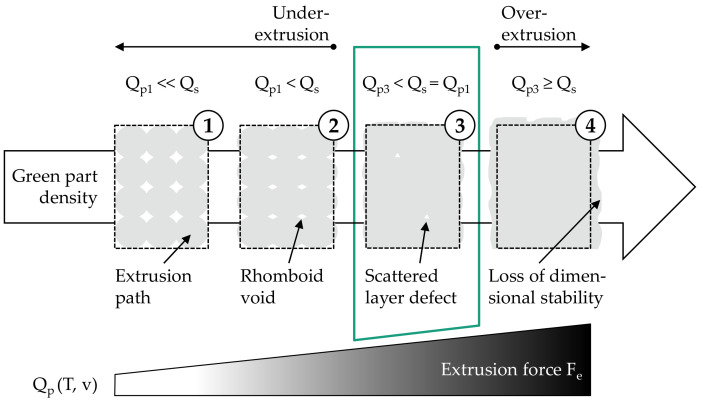
Schematic representation of the influence of the extrusion force on the green part density at a defined printing speed, extrusion temperature and flow rate of 100%.

To print dense green parts resulting in sintered part properties comparable to injection molded parts, under-extrusion must be avoided. In this work, under-extrusion refers to rhomboid voids typical for MEX [30,31], which are created by adjacent extrusion paths due to their elliptical shape (s. Figure 6, states 1 and 2). For PEX, these are present as soon as a too low volume flow (Q_p1_ < Q_s_) is extruded for a defined printing speed, extrusion temperature, and flow rate of 100% due to an insufficient extrusion force. If the extrusion force is too low, too little material is extruded, since the volume flow equilibrium from Equation (4) is not fulfilled. The lack of material is the result of the required compaction of the molten feedstock inside the cylinder, which now takes place during extrusion. With an increase in extrusion force, the required volume flow Q_s_ is extruded at the nozzle. The measurable volume flow Q_p3_ at RT, on the other hand, is lower due to the density difference. For a sufficiently low melt viscosity, however, the rhomboid voids are largely closed at Q_p1_ = Q_s_ and only scattered layer defects remain (cf. [15,16]), as can be seen in state 3 in Figure 6. In order to be able to eliminate the remaining voids completely in theory, a further increase in volume flow by means of the extrusion force in conjunction with a reduction in melt viscosity is required. Yet, as indicated in state 4 in Figure 6, this leads to a significant loss of dimensional stability. Therefore, for processing MIM feedstock with PEX, state 3 must be obtained.

**Figure 7:**
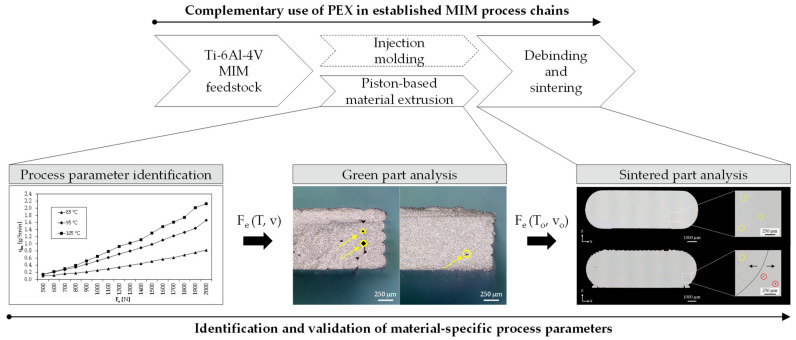
Methodical approach for identification and validation of materials-specific process parameters for a complementary use of PEX in already established MIM process chains.

To achieve state 3, it was assumed that for a given extrusion temperature, printing speed, and a constant flow rate of 100%, an extrusion force could be determined that closes the rhomboid voids while maintaining dimensional stability. For this purpose, suitable extrusion forces had to be derived based on mass flows within the scope of a process parameter identification. The derived extrusions forces as a function of extrusion temperature and printing speed were then evaluated with respect to the green part quality. As a result of the green part analysis, an optimum pair of values for extrusion force, temperature, and printing speed was determined. Finally, the corresponding sintered part quality was evaluated with regard to complementary use in MIM. A schematic summary of the described methodical approach can be found in Figure 7.

#### 2.4.1. Process Parameter Identification

**Figure 8:**
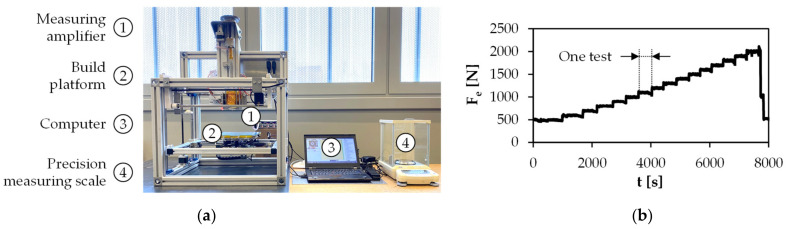
(**a**) Test setup for determination of mass flows; (**b**) exemplary extrusion force plot within the tested force interval.

Within the scope of the process parameter identification, the first step was the experimental determination of mass flows q_me_ as a function of F_e_ and T. For this, a force range of 500 to 2000 N was investigated. On the one hand, this range was intended to ensure the measurement of the smallest amounts of material for a high melt viscosity, and on the other hand, a possible system failure was prevented by limiting the extrusion force to 2000 N. To begin with, the feedstock was filled to at least the middle of the cylinder, followed by moving the piston over the solid material which was then melted for at least 30 min without applying any force. The extrusion force measured at the piston rod was then zeroed and the molten feedstock was manually compacted to 500 N via gear drive and extruded through the nozzle onto the build platform for five minutes at constant extrusion force (s. Figure 8a). In order to ensure a constant force load, F_e_ was plotted via measuring amplifier on the computer and controlled manually, as can be seen in Figure 8b. The extruded material was then weighed using a precision measuring scale (Shimadzu, AUW220D).

To generate the next measuring point, the extrusion force was increased by 100 N and the described approach was repeated for the lowest extrusion temperature until the force limit of 2000 N was reached. For testing the next higher extrusion temperature, the feedstock was again melted for at least 30 min before repeating the described approach. In order to identify a suitable temperature interval, the Ti-6Al-4V feedstock was first tested with a starting temperature of 75 °C, which is located above the solidification point of the paraffin wax [4,32]. A starting temperature was considered to be identified as soon as a mass flow of at least 0.01 g/5 min at 500 N could be measured. Alternatively, the melt viscosity was reduced accordingly by increasing the temperature by 10 °C and the force interval was tested again. The material-specific interval was found as soon as three extrusions temperatures were identified.

q_me_/ρ_RT_ = Q_p3_ ≥ Q_s_ = v∙A (6)

v_c_ ≤ q_me_/(ρ_RT_∙A) (7)

v_c_: Calculated printing speed [mm/s]
q_me_: Experimentally determined mass flow [g/s]

In a second step, corresponding printing speeds v_c_ as a function of F_e_ and T for the determined mass flows were calculated according to Equations (6) and (7). The printing speeds were chosen so that Q_p3_ was greater than or equal to Q_s_, which resulted in over-extrusion analogous to state 4 in Figure 5. Over-extrusion was intentionally forced to compensate for measurement inaccuracies such as the approximated elliptical shape of the extrusion paths (cf. [28]), the calculated feedstock density, and the manually applied extrusion force during the extrusion tests. Therefore, over-extrusion during printing was considered unlikely and, if present, would be equalized after a short time so that Q_p1_ equals Q_s_. The result of the process parameter identification provided values for the extrusion force theoretically associated with dense green parts at a calculated printing speed and set extrusion temperature.

#### 2.4.2. Green Part Analysis

Validation of the determined extrusion forces was performed in the green part analysis. For this, the fracture surfaces of the additively manufactured green parts were examined for rhomboid voids and an extrusion force was identified for an optimum printing speed and extrusion temperature. As a test specimen, a low-complexity part geometry with dimensions of 20 × 20 × 1.1 mm was used. For each extrusion temperature and printing speed, two test specimens were printed side by side in order to be able to exclude a potential influence of the initial over-extrusion on the green part density. Additional slicing parameters are shown in Table 2. After printing, the green parts were analyzed with respect to their density using a digital microscope (Keyence, VHX-5000). As a quality characteristic for green part density, the fracture surface of the samples was examined for rhomboid voids between the extrusion paths.

#### 2.4.3. Sintered Part Analysis

Finally, the resulting sintered part properties regarding part density, yield strength (YS), ultimate tensile strength (UTS), and elongation at break (ε) were evaluated in comparison to injection-molded specimens debound and sintered with the same process parameters. These are empirical values from the material manufacturer that can be expected for debinding and sintering as described in Section 2.3. In addition, the sintered part quality was compared to a standard for MIM-processed Ti-6Al-4V for medical applications ASTM F2885-11 [33].

**Figure 9:**
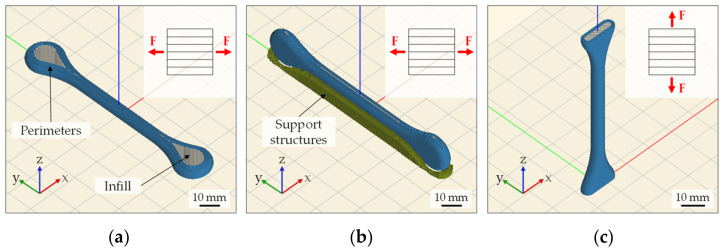
Representation of tensile specimens in Slic3r: (**a**) flat; (**b**) side; (**c**) vertical.

For the sintered part analysis, tensile samples based on DIN EN ISO 2740:2009 [34] were used. Since parts produced by MEX generally exhibit a distinct anisotropy in their mechanical properties [35,36,37], a total of three different build orientations (flat, side, and vertical) were analyzed, as can be seen in Figure 9. The anisotropy is due to the layer bonding which is significantly stronger along the layers than between them [38]. For each build orientation, four tensile specimens were printed. In order to produce the vertically orientated tensile specimens, the specimen heads were cut in half to increase the contact surface to the build platform, as shown in Figure 9c. Otherwise, the specimens would be in danger of tipping over because of the small contact surface in combination with the rapid movements of the build platform.

For the shrinkage analysis, all specimens as both green part and sintered part were measured in x-, y-, and z-direction using a profile projector (Mitutoyo, PJ 3005 TG). Furthermore, Archimedes principle (Mettler Toledo, ME-T with density kit ME-DNY-4) was used to quantify the sintered part density, and micrographs were taken with a digital microscope (Keyence, VHX-5000) to investigate the influence of build orientation. The same microscope was also used for fracture surface analysis of the tensile specimens tested with a universal testing machine (ZwickRoell, Z050).

## 3. Results and Discussion

### 3.1. Process Parameter Identification

**Figure 10:**
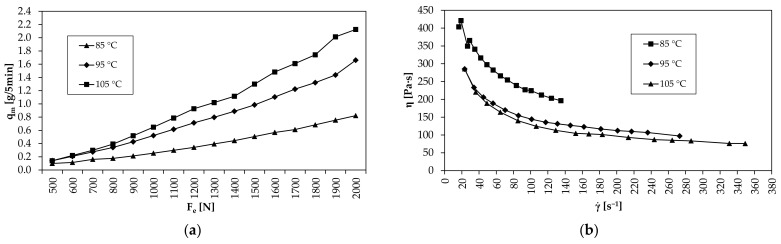
(**a**) Experimentally determined mass flows for tested force and temperature interval; (**b**) viscosity curves showing pseudoplastic flow behavior for tested extrusion temperature interval.

To determine the mass flows q_me_ as a function of F_e_ and T, a temperature interval was first defined. The lower limit of the temperature interval for the Ti-6Al-4V feedstock could be identified at 85 °C, which is close to the solidification point of paraffin wax. The two higher temperatures in this interval were thus to be located at 95 and 105 °C. For all three extrusion temperatures, mass flows were then determined experimentally in the force interval from 500 to 2000 N, as shown in Figure 10a. The viscosity curves Figure 10b derived from the mass flows show a pseudoplastic flow behavior typical for MIM feedstock [2]. Thus, the viscosity starts to decrease significantly as a function of the shear rate. Between the viscosity curves, the influence of the extrusion temperature can also be seen, which results in lower viscosity at the same shear rate [39].

For the determination of the viscosity curves, conventional expressions for shear rate and shear stress in pipe flow are used [40,41]. Both can be determined via the measured mass flows as a function of the extrusion force according to Equations (8) and (9), respectively. However, since MIM feedstock usually exhibits non-Newtonian flow behavior [2], corrections must be made for both shear stress and shear rate by, for example, varying the capillary length while keeping the nozzle diameter constant (Bagley correction [42]). In order not to alter the PEX system design, the apparent viscosity is used in the following, which is calculated according to Equation (10).

τ = (r_n_ ∙ F_e_)/(2 ∙π ∙r_p_^2^ ∙l) (8)

(9) $$\dot{\gamma }=(4\;\cdot \;Qp_{3})/(\pi \;\cdot \;{r_{n}}^{3})$$

(10) $$\eta =\tau /\dot{\gamma }$$

τ: Shear stress [Pa]
r_n_: Nozzle radius [mm]
r_p_: Piston radius [mm]
l: Capillary length [mm]
$\dot{\gamma }$: Shear rate [s^−1^]
η: Viscosity [Pa∙s]

Based on the measured mass flows from Figure 10a, the equivalent printing speed v_c_ was calculated according to Equation (7). The corresponding values are summarized in Table 3. Previously published studies have shown that fast printing speeds decrease the green part density. The maximum possible printing speed of 24 mm/s is thus in a similar range to previously published optimum printing speeds of 20 mm/s [16,17,38]. By applying 4 mm/s increments, the resulting printing speed interval ranges from 4 to 24 mm/s, and those printing speeds that satisfy Equation (6) are highlighted in gray in Table 3. For example, at T = 105 °C and F_e_ = 1100 N, Equation (6) is fulfilled for 8 mm/s, since the calculated printing speed of 9.02 mm/s is higher and thus a dense green part is to be expected. The corresponding over-extrusion is forced to compensate measurement inaccuracies. If, despite measurement inaccuracies, over-extrusion prevails during the printing process, it can be assumed that this will be equalized after a few layers. The reason for this is the manually applied extrusion force during the determination of the mass flows. In the printing process, the extrusion force correlates with the set piston speed and extrusion temperature, so that the initial excessive volume flow automatically approaches the set volume flow equilibrium Q_p1_ = Q_s_.

### 3.2. Green Part Analysis

**Figure 11:**
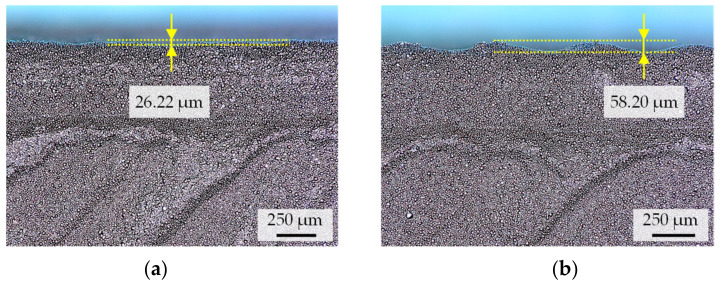
Digital images of the test specimen edge in x-y-plane: (**a**) T = 95 °C, v_c_ = 8 mm/s, F_e_ = 1300 N; (**b**) T = 95 °C, v_c_ = 12 mm/s, F_e_ = 1700 N.

During the printing tests, it was found that the specimens exhibited shape deviations in the form of sinuous waves in the edge region when the printing speed was increased from 8 to 12 mm/s (s. Figure 11). The resulting amplitude magnitude is twice as high as shape deviations due to layer deposition, which can be attributed to the vibrations as a result of the high acceleration values in the firmware. The values were chosen high in order to make the extrusion process as constant as possible, in which acceleration and deceleration phases associated with under- or over-extrusion are reduced to a minimum. Consequently, the maximum printing speed was limited to 8 mm/s in order to exclude the effects of vibrations on the green part density. In this context, it should be noted that green part manufacturing is only part of the production time for one sintered part. Debinding and sintering have a significant influence as well so that only the printing speed is not decisive. In addition, lower printing speeds (5 mm/s) have been documented specifically for the filament-based material extrusion of a Ti-6Al-4V feedstock [43]. Thus, a speed of 8 mm/s is comparable to the state of the art.

**Figure 12:**
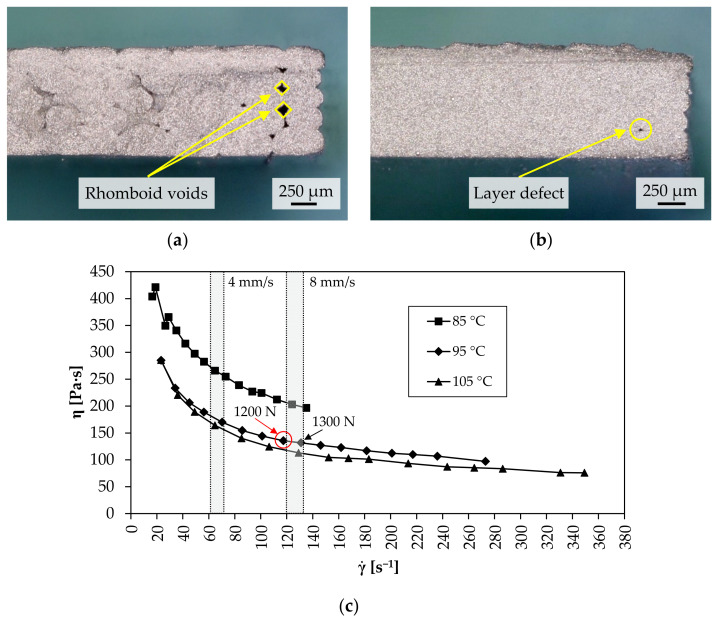
Digital images of fracture surface in z-x-plane of a test specimen printed at v = 8 mm/s and T = 95 °C: (**a**) F_e_ = 1200 N; (**b**) F_e_ = 1300 N; (**c**) viscosity curves with gray highlighted shear rate range for dense green parts at v = 4 mm/s and v = 8 mm/s.

For the remaining printing speed interval, it was found that, with one exception, the calculated v_c_ values provided the correct extrusion force for dense green parts according to state 3 in Figure 6. Only at an extrusion temperature of 95 °C and a printing speed of 8 mm/s did the required extrusion force deviate by 100 N from the F_e_ value derived from Table 3. As shown in Figure 12a, typical rhomboid voids are visible at the outer edge of the corresponding specimen. However, it should be noted that of all the values listed in Table 3, the calculated printing speed value of 8.20 mm/s is the one closest to its threshold (+0.20 mm/s). Thus, it can be concluded that the derived extrusion force from this printing speed value was not sufficient to extrude the required volume flow, which correlates with the shear rate according to Equation (9). One explanation for the deviation could be the additional extrusion force during layer deposition which did not prevail during the determination of mass flows since material extrusion was carried out in air. Due to insufficient force, the shear rate at 1200 N ($\dot{\gamma }\;$= 117 s^−1^) is outside the shear rate range required for dense green parts. As shown in the gray highlighted areas in Figure 12c, the corresponding shear rate range for v = 4 mm/s and v = 8 mm/s is $\dot{\gamma }$ > 60 s^−1^ and $\dot{\gamma }$ > 120 s^−1^, respectively. For the maximum printing speed of 8 mm/s and extrusion temperature of 95 °C, this is only fulfilled with an extrusion force of at least 1300 N ($\dot{\gamma }\;$= 131 s^−1^), which further reduces the viscosity by 4 Pa∙s. Consequently, for processing the Ti-6Al-4V feedstock at 95 °C, an extrusion force must prevail that ensures a viscosity of η ≤ 132 Pa∙s in a shear rate range of $\dot{\gamma }\;\;$> 120 s^−1^. Due to the temperature dependence of the feedstock, the viscosity required to reach this shear rate range decreases (85 °C: η ≤ 203 Pa∙s; 105 °C: η ≤ 113 Pa∙s) and so does the extrusion force (85 °C: F_e_ = 1900 N; 105 °C: F_e_ = 1100 N) with increasing extrusion temperature, as shown in Figure 12c.

**Figure 13:**
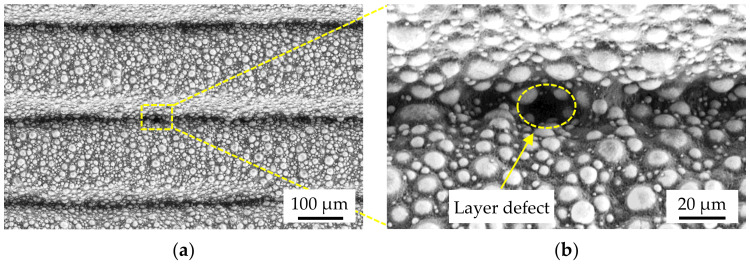
SEM images of a test specimen printed at v = 8 mm/s, T = 95 °C and F_e_ = 1300 N with an exemplary layer defect in the z-x plane at different magnification levels: (**a**) 100×; (**b**) 500×.

Based on the viscosity curves, an increase in the extrusion force from 1200 to 1300 N at 95 °C consequently results in a dense green part. As shown in Figure 12b, only one small void can be seen in the sample, which is due to a layer defect. These can generally be identified between two deposited layers, as shown in the SEM images (Leo Gemini 1530) in Figure 13a,b. These layer defects are not due to under-extrusion, but to the formation of the layer bonding during deposition and are nearly impossible to eliminate [15,16].

Thus, for the chosen printing speed of 8 mm/s, it was possible to methodically determine an extrusion force that prevents under-extrusion for each extrusion temperature tested within the printing speed interval. A temperature of 95 °C (F_e_ = 1300 N) was then selected for validation of the final sintered part properties, as this represents a compromise between good layer bonding (increases with higher extrusion temperatures) and high dimensional stability (decreases with higher extrusion temperatures) within the determined temperature interval [35].

### 3.3. Sintered Part Analysis

**Figure 14:**
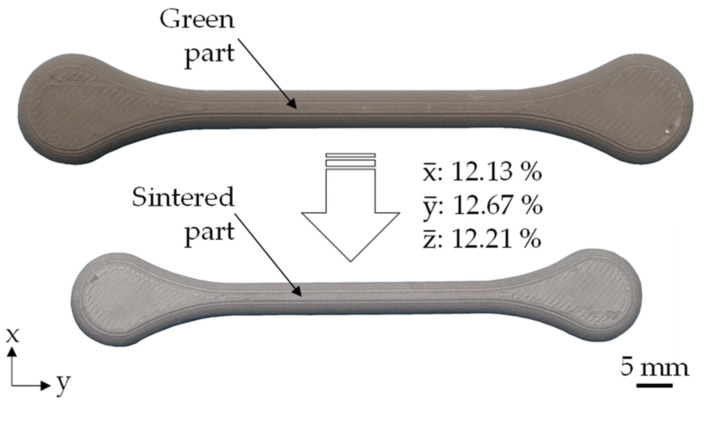
Average shrinkage of the PEX printed tensile specimens due to sintering; shown here is the flat orientation.

The basis for the sintered part analysis was provided by the green parts printed with the optimum process parameters from Section 3.2. To compensate for shrinkage due to sintering, scaling factors similar to those used in injection molding were used and modified so that the part height was equal to the multiple of the layer height. The printed and scaled green parts were then debound and sintered as described in Section 2.3. It was found that there was no significant shrinkage between the green and brown parts after solvent debinding. The shrinkage due to sintering averaged over all printed tensile specimens is 12.13% in x, 12.67% in y, and 12.21% in the z direction (s. Figure 14). Between the three selected build orientations, there is a maximum difference in shrinkage of 0.13% in the x, 0.50% in the y, and 0.58% in the z direction, which is most likely caused by the build orientation and the associated geometry changes. Thus, in contrast to MIM, the shrinkage is anisotropic, which is due to the layer-based additive manufacturing process (cf. [44]). In addition, the part geometry has a major influence on shrinkage [45,46], which is determined empirically for each mold in MIM to ensure dimensional accuracy [47]. Compared to the MIM reference, the shrinkage of the PEX printed specimens is generally lower, with a maximum deviation in the x direction of 0.87% on average. It is assumed that the lower shrinkage is a combined effect of geometry changes due to the build orientation and the process parameters prevailed during green part printing [25].

Therefore, in order to increase the dimensional accuracy for near-net-shape part production with PEX, scaling factors must be determined for each geometry. In this context, it is important to ensure that the green part density is homogeneous and that the printing direction defines the debinding and sintering orientation. Additional factors that influence sintering shrinkage include, for example, the friction between baseplate and part, the bending of unsupported features, or gravity [44]. All these factors have to be considered and combined in a geometry-specific scaling factor. This can be determined either empirically on the basis of test geometries or with the aid of simulation software (cf. [48]).

**Figure 15:**
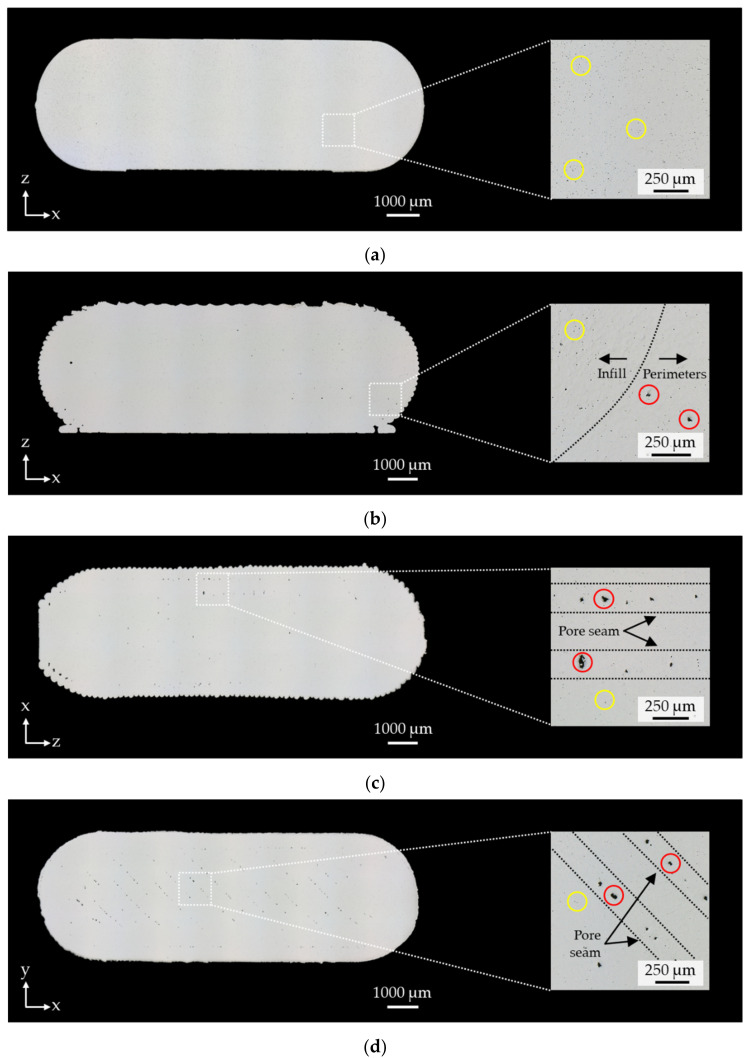
Micrographs of the cut specimen heads from the MIM reference and the investigated build orientations for density analysis; pores due to sintering marked in yellow and pores due to PEX marked in red: (**a**) MIM reference; (**b**) flat orientation; (**c**) side orientation; (**d**) vertical orientation.

Furthermore, a mandatory prerequisite for the integration of PEX into established debinding and sintering process chains is a sufficiently high density in the sintered part. As can be seen in Table 4, the density values for PEX printed and sintered parts range on average between 98.4% and 99.1% of the theoretical density which is in the range of injection-molded specimens (99%) and above the MIM standard ASTM F2885-11 (min. 96%). Figure 15 shows that the MIM reference sample has very fine pores located in a range < 5µm. In addition to the pores caused by sintering, the PEX printed samples show large pores in a range of <50 µm. These pores are due to layer defects and are mainly located in areas related to the respective build orientation. Thus, the PEX-related large pores in the flat orientation can be primarily identified between the outer contour lines (perimeters) in the lower region. Here, the perimeters sag slightly due to the specimen geometry, resulting in pores >5 µm. These can also be seen in the side orientation. It is noticeable that the porosity is mainly in the middle area of the specimen in the form of pore seams. This is most likely due to the support structure that was required for printing (s. Figure 9b). For this purpose, a gap of 0.2 mm between the support structure and the specimen is provided by the slicing software in order to be able to remove both from each other again before debinding. However, this leads to sagging of the corresponding layers, which is compensated for as the number of layers increases. The sagging leads to a local weakening of the layer bonding, resulting in layer defects in the green part and thus pores in the sintered part. An examination of the x-y plane in Figure 15d also shows that PEX-related pores are always present between the extrusion paths, which is independent of the build orientation. The resulting pore seams can especially be seen in the x-y plane due to the ±45° filing pattern. However, the 0.7% lower average density for the vertical-orientated compared to the flat-oriented samples is most likely due to the path planning of the slicing software. Due to approximation errors, circular features can only be filled with 100% infill to a limited extent. This is especially true for the circular center section of the vertically oriented specimens resulting in higher porosity in the test area. Yet, the resulting residual porosity is only 1.6%, which is still above typical values for titanium injection molding (between 3 and 4% [28]) and similar to the reference density value in Table 4.

**Figure 16:**
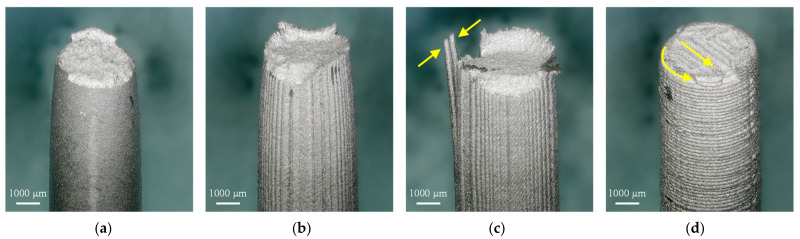
Fracture surfaces of tested tensile specimens (**a**) MIM reference; (**b**) flat orientation; (**c**) side orientation, yellow arrows show poorly connected extrusion paths in the area of the support structure; (**d**) vertical orientation, yellow arrows highlight visible extrusion paths in the fracture surface.

As shown in Figure 16a, the MIM reference exhibits a ductile overload fracture with the characteristic cup and cone fracture surface. A similar fracture failure can be observed for the tensile specimens for the flat orientation, characterized by a slightly higher yield strength (+33 MPa) and lower elongation at break (−1.5%). The small differences are most likely due to the layer-by-layer extrusion process and the resulting large pores visible in the micrographs in Figure 15b. Despite these pores, tensile properties with regard to YS, UTS, and ε could be realized which are in accordance with ASTM F2885-11. This also applies to the side orientation, although the local weakening of the layer bonding as a result of the required support structures led to poorer tensile properties overall. As indicated by the yellow arrows in Figure 16c, few extrusion paths are thus barely connected in the lower areas, which has induced earlier failure. Compared to the flat-orientated specimens, this resulted in a decrease of YS by 102 MPa, UTS by 43 MPa, and ε by 8.4%. The fracture surface, however, exhibits ductile overload failure with the characteristic cup and fracture surface, as already observed for the MIM reference and the flat-orientated specimens. This is different from the vertical-orientated specimens. Here, the overall lowest mean value for elongation at break was measured at 3.4%, which is outside the ASTM F2885-11 standard and can be attributed to the force loading between the layers.

**Figure 17:**
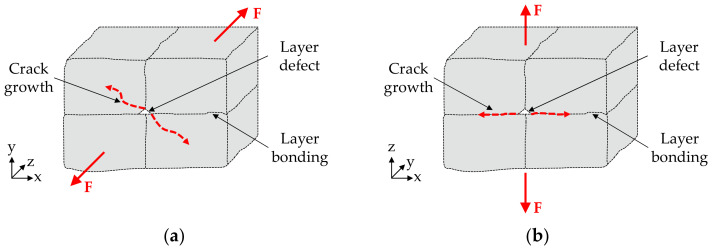
Schematic representation of fracture model (cf. [38]): (**a**) Flat orientation: crack growth within extrusion paths; (**b**) vertical orientation: crack growth between the layer bonding.

Thus, crack growth took place between the layer bonding and not within the deposited extrusion paths, as shown schematically in Figure 17. This is due to the pore seams between the extrusion paths (s. Figure 15d) that weaken the layer bonding and are only subjected to tensile stress in the vertical-orientated specimens resulting in a brittle overload fracture. The planar fracture surface characteristic of this fracture behavior can be seen in Figure 16d, in which extrusion paths are visible (yellow arrows).

Consequently, it was possible to achieve comparable properties in the sintered part for the flat-oriented specimens using the methodically derived process parameters for green part production. Therefore, the green parts should be positioned accordingly on the build platform for complementary use with regard to tensile properties and part density. Otherwise, there is a significant decrease in both, which is especially true for the vertically oriented samples.

## 4. Conclusions

In this work, an industrially used Ti-6Al-4V MIM feedstock was processed with piston-based material extrusion with the aim of producing sufficiently dense green parts to enable complementary use of PEX in already established MIM process chains. For this, material-specific process parameters were methodically derived on the basis of the extrusion force. It was found that an extrusion force of 1300 N at a maximum printing speed of 8 mm/s in combination with an extrusion temperature of 95 °C resulted in sufficiently dense green parts. These material-specific process parameters were then used to print test specimens for comparison with MIM. It was found that density values only deviate from the MIM reference by a maximum of 0.6%, which is still 2.4% above the minimum of ASTM F2885-11. Furthermore, it was shown that the build orientation has a decisive influence on the tensile properties, which can be attributed to the PEX-related pores. However, flat orientated test specimens deviate only slightly from the MIM reference (YS: +33 MPa, UTS: 0 MPa, ε: −1.5%) and also meet ASTM F2885-11 with regard to minimum tensile properties. Thus, a complementary use could be proven in principle for the Ti-6Al-4V MIM feedstock. However, it will be the subject of further research to accurately predict shrinkage of PEX printed green parts in order to ensure dimensional accuracy for different geometries.

## Figures and Tables

**Table 1:** Chemical composition of used Ti-6Al-4V feedstock as-sintered [25], reproduced with permission from Element22 GmbH.

Element	Ti	Al	V	C	N	Fe	O	H	Y
wt.%	Balance	5.5–6.75	3.5–4.5	≤0.045	≤0.035	≤0.30	≤0.30	≤0.015	≤0.005

**Table 2:** Used printing parameters set in Slic3r and corresponding representation of test specimen.

Slicing Parameters	Values	Test Specimen
Nozzle diameter	0.40 mm	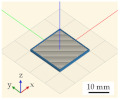
Layer height	0.20 mm	
Track width	0.45 mm	
Flow rate	100%	
Infill density	100%	
Infill pattern	±45°	
Bed temperature	60 °C	

**Table 3:** Calculated printing speeds (v_c_) as a function of F_e_ and T; v_c_ values that satisfy Equation (6) are highlighted in gray.

T [°C]	F_e_ [N]															
	500	600	700	800	900	1000	1100	1200	1300	1400	1500	1600	1700	1800	1900	2000
85	1.15	1.32	1.85	2.03	2.45	2.93	3.43	3.93	4.52	5.09	5.81	6.52	7.02	7.85	8.66	9.42
95	1.62	2.38	3.14	3.92	4.90	5.99	7.06	8.20	9.14	10.20	11.30	12.67	14.02	15.16	16.47	19.06
105	1.62	2.52	3.43	4.51	5.95	7.43	9.02	10.63	11.69	12.79	14.91	17.00	18.46	19.99	23.09	24.38

**Table 4:** Comparison of the additively manufactured specimens in orientation flat, side, and vertical with ASTM F2885-11 and MIM reference in terms of part density and tensile properties.

Specimens	Density [%]	Tensile Properties		
		YS [MPa]	UTS [MPa]	ε [%]
ASTM F2885-11	min. 96 ^1^	min. 680	min. 780	min. 10
MIM reference	99	900	1000	20
Flat	99.1	933	1000	18.5
Side	98.8	831	957	10.1
Vertical	98.4	866	968	3.4

^1^ as-sintered.

## Data Availability

The data presented in this study are available on reasonable request from the corresponding author.
